# Does this lung nodule need urgent review? A discrete choice experiment of Australian general practitioners

**DOI:** 10.1186/s12890-020-1053-x

**Published:** 2020-01-30

**Authors:** P. Brownell, F. Piccolo, F. Brims, R. Norman, D. Manners

**Affiliations:** 1 Department of Respiratory Medicine, St John of God Healthcare Midland Campus, Midland, Western Australia; 2 Department of Respiratory Medicine, Sir Charles Gairdner Hospital, Nedlands, Western Australia; 3 Curtin University Medical School, Bentley, Western Australia; 4 Curtin University School of Public Health, Bentley, Western Australia

**Keywords:** Lung cancer, Lung nodule, General practitioners, Referral patterns, PanCan model

## Abstract

**Background:**

Lung cancer is the leading cause of cancer mortality in Australia. Guidelines suggest that patients with suspected lung cancer on thoracic imaging be referred for urgent specialist review. However, the term “suspected” is broad and includes the common finding of lung nodules, which often require periodic surveillance rather than urgent invasive investigation. The British Thoracic Society recommends that a lung nodule with a PanCan risk > 10% be considered for invasive investigation. This study aimed to assess which factors influence general practitioners (GPs) to request urgent review for a lung nodule and if these factors concur with PanCan risk prediction model variables.

**Methods:**

A discrete choice experiment was developed that produced 32 individual case vignettes. Each vignette contained eight variables, four of which form the parsimonious PanCan risk prediction model. Two additional vignettes were created that addressed haemoptysis with a normal chest computed tomography (CT) scan and isolated mediastinal lymphadenopathy. The survey was distributed to 4160 randomly selected Australian GPs and they were asked if the patients in the vignettes required urgent (less than two weeks) specialist review. Multivariate logistic regression identified factors associated with request for urgent review.

**Results:**

Completed surveys were received from 3.7% of participants, providing 152 surveys (1216 case vignettes) for analysis. The factors associated with request for urgent review were nodule spiculation (adj-OR 5.57, 95% CI 3.88–7.99, *p* < 0.0001), larger nodule size, presentation with haemoptysis (adj-OR 4.79, 95% CI 3.05–7.52, *p* < 0.0001) or weight loss (adj-OR 4.87, 95% CI 3.13–7.59, *p* < 0.0001), recommendation for urgent review by the reporting radiologist (adj-OR 4.68, 95% CI 2.86–7.65, p < 0.0001) and female GP gender (adj-OR 1.87, 95% CI 1.36–2.56, p 0.0001). In low risk lung nodules (PanCan risk < 10%), there was significant variability in perceived sense of urgency. Most GPs (83%) felt that a patient with haemoptysis and a normal chest CT scan did not require urgent specialist review but that a patient with isolated mediastinal lymphadenopathy did (75%).

**Conclusion:**

Future lung cancer investigation pathways may benefit from the addition of a risk prediction model to reduce variations in referral behavior for low risk lung nodules.

## Background

Lung cancer is the fifth most common cancer in Australia but is the leading cause of both cancer related mortality and morbidity [1, 2]. Smoking rates continue to fall but the incidence of lung cancer has not yet plateaued and the five-year survival remains below 20% [1, 2].

Given the poor outcomes for many lung cancer patients and the ongoing costs to the healthcare system, best practice guidelines have been created to streamline and standardise the lung cancer diagnosis and treatment pathway. These pathways emphasise timely review and early involvement of a lung cancer specialist [3, 4]. Both the Australian Optimal Care Pathway (OCP) and the British guideline recommend review by a specialist within two weeks of referral following thoracic imaging suspicious of lung cancer [4, 5]. The lung cancer flowchart endorsed by the Royal Australian College of General Practitioners (RACGP) recommends that any suspicious findings on chest computed tomography (CT) or any new or changing lung nodule receive an urgent specialist referral [3, 4].

The term “suspected” lung cancer is used in both Australian pathways but is not clearly defined and thus may incorporate lung nodules, where the risk of malignancy is low and the need for specialist review may not be urgent. A lung nodule is defined as a rounded or irregular opacity, measuring less than 30 mm in diameter [6]. With the widespread availability of CT scanning in Australia, the finding of lung nodules is increasingly common, with the reported prevalence being almost 9% in a Western Australian cohort [7]. However, the rate of cancer in screen-detected lung nodules was only 5.5 and 3.7% in two high risk Canadian cohorts respectively and is likely to be even lower in an unselected population [8]. The appropriate management of many lung nodules is serial imaging many months apart to assess nodule growth [9].

Both local and international research suggests that adhering to the suggested timeframes for urgent review of suspected lung cancer can be challenging. An audit of the respiratory service at our hospital several years ago demonstrated that 73% of suspected lung cancer referrals were reviewed within two weeks [10]. However, the most recent scoping review on the topic suggests variability in review times, ranging from 0 to 33 days across 23 countries, compared to the recommended 14 days [11]. The case-mix of a typical respiratory service has not been documented but the high prevalence of lung nodules and their inclusion in suspected lung cancer referral pathways dictates that a meaningful proportion of the workload will be attributable to lung nodules with low malignancy risk.

The PanCan (or Brock University) model is a probabilistic risk-based model that calculates the malignancy risk of a pulmonary nodule detected on first screening low dose CT scan, using both radiographic and patient characteristics. The model has an area under the curve of more than 0.90 and shows excellent predictive accuracy for lung cancer even in small nodules [8]. The British Thoracic Society (BTS) guidelines on investigation and management of lung nodules suggest that a nodule with a PanCan risk > 10% should be considered for invasive investigation [9].

In view of the high prevalence of lung nodules, the overall low cancer risk and the lack of clarity in published pathways surrounding the need for urgent specialist review, this study was designed to assess the referral behaviors of Australian general practitioners (GPs) using a discrete choice experiment (DCE) approach.

## Methods

### Aims

1. To identify factors that influence GPs to request urgent specialist review for patients with a lung nodule on CT scan.
2. To assess the proportion of GP responses that request urgent review for lung nodules with a PanCan risk > 10%.
3. To assess the proportion of GPs requesting urgent review in the specific clinical scenarios of a) A patient with haemoptysis and a normal chest CT and b) A patient with mediastinal and hilar lymphadenopathy without a parenchymal lung lesion.

### Definitions and rationale

Urgent review was defined as review by a lung cancer specialist within two weeks of referral for suspected lung cancer.

The BTS recommendation that a lung nodule with a PanCan risk > 10% be considered for further investigation with positron emission tomography (PET) scan and / or biopsy was used as a surrogate for needing urgent (within two weeks) specialist review.

### Survey development

An orthogonal main effects plan (OMEP) was used to create 32 individual lung nodule case vignettes. An OMEP is a set of combinations of dimensions and levels where, for dimension, the number of times each pair of levels appear is constant. Therefore, for scenarios where (for example) lung nodule spiculation is observed, the location of the nodule is split equally between upper lobe and not upper lobe. Each vignette included eight variables, four of which form the parsimonious PanCan risk prediction model (gender, nodule size, location and spiculation) [8]. The study team developed a further four variables (age, smoking status, symptoms, recommendation from reporting radiologist), which could plausibly be thought to increase lung cancer risk and influence GP sense of urgency (see Table 1). The individual vignettes were then manually entered into Qualtrics (Qualtrics, Provo, UT, USA). Qualtrics is an online survey platform that allows survey development, dissemination and analysis, although only the dissemination feature was used in this study. Each GP was presented with eight randomly selected case vignettes and asked if the patient required urgent (within two weeks) specialist review. An example case vignette is provided in Table 2 and all 32 vignettes are provided as Additional file 1. Vignettes with a parsimonious PanCan risk of > 10% were deemed to require urgent review.

**Table 1:** Vignette variables and response options

Lung nodule case vignette	
Your patient is a 50 year old man. He is a current, lifelong smoker. He has a cough and worsening breathlessness. A CT of his chest shows a 4mm left upper lobe nodule with spiculation. There is no recommendation provided by the reporting radiologist. *Does he need to be seen by a respiratory physician urgently (<2 weeks) for suspected lung cancer?*	
Haemoptysis case vignette	
Your patient is a 60 year old man. He has never smoked. He has a small amount of haemoptysis. A CT of his chest is normal. There is no recommendation provided by the reporting radiologist. *Does he need to be seen by a respiratory physician urgently (<2 weeks) for suspected lung cancer?*	
Lymphadenopathy case vignette	
Your patient is a 70 year old woman. She quit smoking 5 years ago. She has a cough and worsening breathlessness. A CT of her chest shows enlarged subcarinal and hilar lymph nodes without a lung lesion. There is no recommendation provided by the reporting radiologist. *Does she need to be seen by a respiratory physician urgently (<2 weeks) for suspected lung cancer?*

**Table 2:** Case vignettes

Gender, n(%)	
Male	60 (39)
Female	92 (61)
Age, n(%)	
<35 years	20 (13)
35-44 years	29 (19)
45-54 years	42 (28)
55-64 years	31 (20)
65-74 years	26 (17)
>75 years	4 (3)
GP role, n(%)	
Vocationally registered	130 (86)
Non-vocationally registered	11 (7)
Registrar	9 (6)
Other	2 (1)
Years worked in general practice, n(%)	
<5	24 (16)
5-9	23 (15)
10-19	30 (20)
20-29	29 (19)
30-39	28 (18)
>40	18 (12)
Average number of hours worked per week, n(%)	
<20	28 (19)
21-30	32 (21)
31-40	58 (38)
>40	34 (22)
Location of primary practice, n(%)	
Capital city	70 (46)
Other metropolitan area^a^	28 (19)
Rural area^b^	40 (26)
Remote area^c^	14 (9)

^a^Population >100 000, ^b^Population 10 000 – 100 000, ^c^Population <10 000

Each GP was also presented with two additional case vignettes (both provided in Table 2). One described a patient with haemoptysis and a normal chest CT and the other described a patient with mediastinal and hilar lymphadenopathy without a parenchymal lung lesion. These were included because they are not specifically addressed in lung cancer pathways but could represent lung cancer.

Each GP provided demographic information, such as age, gender, location of primary practice, years of experience and number of hours worked each week.

### Survey distribution

The survey was distributed to 4160 randomly selected GPs throughout Australia via email, with the use of the Australasian Medical Publishing Company (AMPCo) practitioner database. The email invite included some background information about the Cancer Council OCP and a link to the Qualtrics survey platform, where the GP would be presented with eight randomly selected case vignettes, as well as the additional vignettes regarding haemoptysis and lymphadenopathy. Consent was implied if the survey was completed. Reminder emails were sent at two and four weeks. Participants had the option of providing their contact details to win one of five $100 incentive vouchers. Ethics approval for the study was granted by the St John of God Human Research Ethics Committee (reference 1302).

### Statistical analysis

A multivariate logistic regression model was used to identify factors significantly associated with request for urgent review that initially included all vignette and demographic variables and then applied stepwise exclusion of factors with *p* values > 0.05. Ordinal vignette and demographic variables (nodule size, patient age and hours worked per week) were considered as continuous independent variables for the model. Statistical analysis was performed using the SAS university edition with SAS Studio version 3.8 (SAS Institute Inc., Cary, NC, USA).

## Results

A total of 4160 surveys were distributed and 157 GPs began the survey, giving a response rate of 3.8%. Five participants did not complete the survey and were excluded from further analysis, giving a final sample size of 152 GPs (3.7%). The participants’ demographic information is described in Table 3. Almost two thirds of respondents were female (61%). The majority were vocationally registered GPs (86%). Almost half (46%) worked in a capital city and a quarter (26%) worked in a rural area.

**Table 3:** Participant demographic information, *n* = 152

Gender, n(%)	
Male	60 (39)
Female	92 (61)
Age, n(%)	
< 35 years	20 (13)
35–44 years	29 (19)
45–54 years	42 (28)
55–64 years	31 (20)
65–74 years	26 (17)
> 75 years	4 (3)
GP role, n(%)	
Vocationally registered	130 (86)
Non-vocationally registered	11 (7)
Registrar	9 (6)
Other	2 (1)
Years worked in general practice, n(%)	
< 5	24 (16)
5–9	23 (15)
10–19	30 (20)
20–29	29 (19)
30–39	28 (18)
> 40	18 (12)
Average number of hours worked per week, n(%)	
< 20	28 (19)
21–30	32 (21)
31–40	58 (38)
> 40	34 (22)
Location of primary practice, n(%)	
Capital city	70 (46)
Other metropolitan area*	28 (19)
Rural area^#^	40 (26)
Remote area^	14 (9)

*Population > 100,000 ^*#*^ *Population 10,000–100,000*

^*^*^ *Population < 10,000*

The factors associated with request for urgent review are summarised in Table 4. The most significant was presence of nodule spiculation, with a spiculated lung nodule 5.57 times more likely to garner a request for urgent review than a non-spiculated nodule (95% CI 3.88–7.99, *p* < 0.0001). Increasing nodule size was also associated with request for urgent review (adj-OR 1.35 per increasing size increment, 95% CI 1.25–1.45, *p* < 0.0001), as was recommended urgent respiratory review from the reporting radiologist (adj-OR 4.68, 95% CI 2.86–7.65, p < 0.0001). Patient factors associated with request for urgent review included being a current smoker (adj-OR 2.90, 95% CI 1.78–4.72, p < 0.0001 compared with a never smoker) and presenting with haemoptysis or unintentional weight loss (adj-OR 4.79, 95% CI 3.05–7.52, p 0.0003 and adj-OR 4.87, 95% CI 3.13–7.59, p 0.0001 respectively, compared with a lung nodule found incidentally). Increasing patient age made request for urgent review less likely (adj-OR 0.78 per increasing age interval of ten years, 95% CI 0.67–0.91, p 0.001).

**Table 4:** Factors associated with request for urgent review

Variable	Adj-OR	95% CI	*p* value
Patient age (per increasing 10 year increment)	0.78	0.67-0.91	0.001
Smoking history			
• Never smoker	Reference		
• Current smoker	2.90	1.78-4.72	<0.0001
• Recent ex-smoker	1.37	0.87-2.17	0.86
• Distant ex-smoker	0.99	0.63-1.55	0.01
Respiratory symptoms			
• Incidentally found (no symptoms)	Reference		
• Cough and breathlessness	2.70	1.76-4.15	0.76
• Haemoptysis	4.79	3.05-7.52	0.0003
• Unintentional loss of weight	4.87	3.13-7.59	0.0001
Nodule spiculation (yes vs no)	5.57	3.88-7.99	<0.0001
Nodule size (per increasing size increment)	1.35	1.25-1.45	<0.0001
Radiologist recommendation			
• No recommendation provided	Reference		
• Specialist respiratory review	2.22	1.41-3.48	0.01
• Urgent respiratory review	4.68	2.86-7.65	<0.0001
• Repeat CT as per guidelines	0.56	0.36-0.87	<0.0001
GP gender (female vs male)	1.87	1.36-2.56	0.0001
Hours worked per week by GP (per increasing interval)	0.83	0.71-0.97	0.02
*Alpha intercept 1.09*			

Female GPs were almost twice as likely to request urgent review as male GPs (adj-OR 1.87, 95% CI 1.36–2.56, p 0.0001) and GPs who worked more hours per week were less likely to request urgent review (adj-OR 0.83 per increasing interval of hours worked per week, 95% CI 0.71–0.97, p 0.015).

**Fig. 1:**
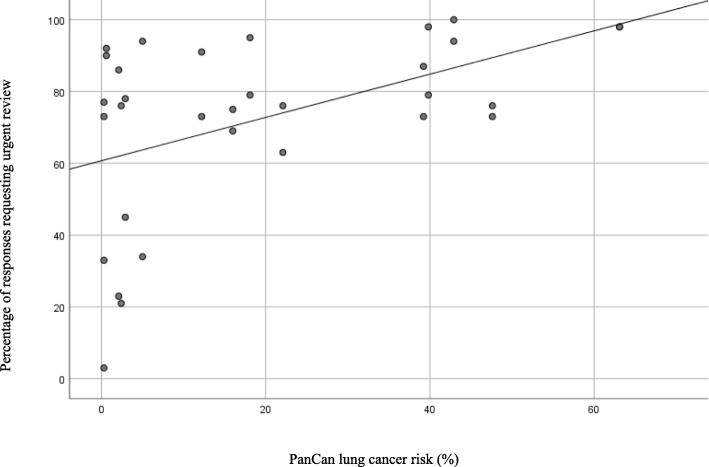
Relationship between PanCan risk and GP perception of urgency***.*** *Legend: Correlation coefficient 0.484, p 0.005*

From the 1216 vignettes analysed, almost two thirds of responses (65%) were concordant with the BTS guidelines and correctly identified high or low risk lung nodules. The relationship between PanCan risk and the proportion of responses requesting urgent review is presented in Fig. 1. In low risk lung nodule vignettes, there was significant variation in the request for urgent review, ranging from 3 to 94% of responses for individual vignettes. In lung nodule vignettes with a PanCan risk > 10%, there was a more consistent request for urgent review, ranging from 63 to 100%.

When asked if a patient with haemoptysis and a normal chest CT needed to be seen by a specialist within two weeks, most GPs answered “No” (83%). When asked the same question of a patient with mediastinal and hilar lymphadenopathy but no parenchymal lung lesion, three quarters answered “Yes” (75%).

## Discussion

This study has examined for the first time the referral patterns for lung nodules amongst a cohort of Australian GPs and identified factors that influence a perceived need for urgency. There is evidence of wide variation in practice, presumably due to interpretation of nodule risk, as well as participant characteristics (such as gender and number of hours worked per week).

Some of the factors associated with request for urgent review in this study were in keeping with recommended practice, whilst others were not. Table 5 summarises these factors and compares them to predictors of malignancy in three validated lung nodule risk prediction models [8, 12, 13]. GPs tended to overestimate the significance of nodule spiculation and underestimate the impact of increasing patient age, female gender and nodule upper lobe location.

**Table 5:** Factors associated with request for urgent review compared with adjusted odds ratios for lung cancer risk factors in validated risk prediction models

	*Current study*	*Parsimonious PanCan model* [8]	*Mayo model* [12]	*Veterans Administration model* [13]
Patient age (per increasing ten year increment)	0.78	Not included	2.72	2.20
Patient gender, female vs male	Not significant	1.91	Not included	Not included
Smoking history	2.90 for current smokers	Not included	2.21 for current smokers	7.90 for current or ex-smokers
Nodule spiculation	5.57	2.54	2.83	Not included
Nodule diameter	1.35 per increasing size increment	Non-linear relationship with cancer risk	1.14 per mm increase in size	1.10 per mm increase in size
Upper lobe location	Not significant	1.82	2.19	Not included

This study has demonstrated that the radiology report and recommendation of the reporting radiologist has a significant impact on GP referral behavior and this finding has also been seen in other literature. Blagev et al. reviewed 1000 CT pulmonary angiogram reports and found that appropriate follow up of incidental pulmonary nodules increased from 0 to 29%, when the reporting radiologist provided an overt suggestion in the conclusion of the report, compared with the nodule only being mentioned in the body of the report [14]. Similar results were found by Woloshin et al where requests for PET scan, serial CT imaging and lung biopsy were compared between two groups of physicians, with one group receiving a standard radiology report and the other receiving an enhanced radiology report [15]. The enhanced radiology report included the lung cancer risk and an explicit recommendation for management, according to the Fleischner Society guidelines and resulted in significantly more clinicians choosing the correct management strategy. It can be seen that the radiology report has a significant effect on subsequent patient management and this has implications for patient care and resource usage when lung nodules are reported in an unstandardized and haphazard fashion. The American College of Radiology uses a standardised tool to report screen-detected lung nodules. The Lung CT Screening Reporting and Data System (Lung-RADS) classifies lung nodules according to size, growth and CT appearance and calculates malignancy risk (using the PanCan risk prediction model) and provides a recommendation for management [16]. A similar process for standardised lung nodule reporting is not currently used in Australia.

Female GPs were almost twice as likely to request urgent review as their male counterparts and this finding has been seen to a lesser extent in Canadian literature. Three papers found that female primary care physicians made 8% more referrals and were 12 and 15% more likely to refer than males [17–19]. The significance of the finding in the present study is not apparent but previous papers have discussed whether this gender disparity represents more appropriate management by female physicians or excessive use of investigations and healthcare resources [19].

Two specific and clinically important scenarios that are not addressed in the Australian Cancer Council OCP or the GP investigation pathway are patients with haemoptysis and a normal chest CT and patients with mediastinal lymphadenopathy without a parenchymal lung lesion. Most GPs surveyed did not think a patient with small volume haemoptysis and a normal chest CT required urgent specialist review and this is in accordance with both local and international guidelines [20]. The haemoptysis Diagnostic Imaging Pathway that is endorsed by the Western Australian Department of Health only suggests CT chest in selected high risk patients and bronchoscopy only when CT does not identify a cause for symptoms [20]. This conservative approach to haemoptysis management is based on several studies that have shown the additional diagnostic yield for lung cancer from bronchoscopy is very low when thoracic imaging has not already demonstrated concerning findings [21–23]. The appropriate management of mediastinal and hilar lymphadenopathy without a parenchymal lung lesion is less clear. Isolated mediastinal and hilar lymphadenopathy (IMHL) is common and the prevalence has been reported as 1–6% [24]. The causes of IMHL include reactive, granulomatous, infectious and malignant diseases [25]. Malignancy accounts for only 13% overall [25]. The American College of Radiology guideline on the management of chest CT incidental findings acknowledges that robust literature on IMHL is lacking but recommends that any patient with a mediastinal lymph node ≥15 mm in short axis without an obvious clinical or radiological explanation, be referred for specialist consultation [26]. In this current study, 75% of GPs thought a patient with IMHL required urgent specialist review and it is reasonable for these patients to be referred to a respiratory specialist, given the complexity and heterogeneity of the condition. However, the risk of cancer is low and review may not need to be within two weeks.

Overall, this study found more variation in responses in lung nodules with a PanCan risk < 10%, with up to 94% of responses still requesting urgent review of these low risk nodules. This may be because the investigation pathway recommended for Australian GPs suggests urgent review in any new or changing nodule, regardless of other clinical or imaging risk factors [3]. Lung nodules with a PanCan risk > 10% yielded a more consistent request for urgent specialist review. Of the 1216 vignettes analysed, 521 had a PanCan risk < 10%, however urgent review was still requested in over half of these (307 vignettes). Cancer investigation pathways are deliberately broad to minimise missed cases but the addition of a risk prediction model to the lung cancer pathway may help to streamline the process. As has been described, the ability of specialist respiratory services to meet the recommended timeframes for review of suspected lung cancer is variable and any measures to alleviate pressure would be worth considering. To the best of our knowledge, risk prediction models are not included in any international lung cancer referral pathways, so their potential impact on referral rates or review times is unknown. While most of the lung cancer risk prediction models were developed in lung cancer screening cohorts, the PanCan risk prediction model has recently been validated in a heterogenous Dutch population and still been found to perform well, with an area under the curve of 0.90 and 0.91 in two separate cohorts [27]. This goes further toward confirming its appropriateness for use in a general population.

There are a number of limitations that should be considered in interpretation of this study. The low response rate (3.7%) may have led to selection bias, although participating GPs were representative of all the Australian States and Territories, except the Northern Territory. Response rates to physician surveys are often poor and have been reported to be declining, due to a number of factors, including lack of time, ineligibility and inaccuracy in registration details [28, 29]. Interestingly, more than one third of practices had a policy not to respond to survey requests in one Canadian study [28]. The choice to use AMPCo was based on a 2017 survey of Australian medical practitioners that used similar distribution methodology and garnered a response rate of 17.5% [30]. Unfortunately, our response rate was much lower, however, the large number of vignettes for analysis is an advantage and does increase the generalisibility of the results. Lung cancer risk was calculated using the parsimonious PanCan risk prediction model, instead of the full version of the model. The BTS guidelines reference the full version, however this includes an additional five variables (age, family history of lung cancer, emphysema, nodule type and nodule count) and it was felt that would be too onerous for study participants. Furthermore, McWilliams et al. found excellent discrimination in both the parsimonious and full versions of the model in their 2013 paper [8]. Finally, the inherent risk of artifice when using case vignettes should be considered. In the interests of brevity, only eight variables were included in the vignettes but real world patients are much more complex and it is certainly possible that some subtleties in referral factors and behaviors have been overlooked.

## Conclusion

This study demonstrates variability in the sense of urgency for referral for lung nodules and highlights that most concern is driven by nodule spiculation, patient presentation with haemoptysis or weight loss and the recommendation of the reporting radiologist. Standardised reporting of lung nodules by radiologists and the addition of estimated nodule risk to lung cancer investigation pathways may help to ensure an effective, timely patient journey from chest CT to specialist review and ensure that all patients are being managed in a more evidence-based fashion.

## Supplementary information

**Additional file 1.** Lung nodule case vignettes.

## Data Availability

The data set analysed in this study is available from the corresponding author upon reasonable request.

## Abbreviations

Adj-OR: Adjusted odds ratio
AMPCo: Australasian Medical Publishing Company
BTS: British Thoracic Society
CI: Confidence interval
CT: Computed tomography
CTCA: Computed tomography coronary angiogram
DCE: Discrete choice experiment
GP: General practitioner
IMHL: Isolated mediastinal and hilar lymphadenopathy
OCP: Optimal care pathway
OMEP: Orthogonal main effects plan
OR: Odds ratio
PET: Positron emission tomography scan
RACGP: Royal Australian College of General Practitioners
VA: Veterans Administration
