# Correction to: Does this lung nodule need urgent review? A discrete choice experiment of Australian general practitioners

**DOI:** 10.1186/s12890-020-1085-2

**Published:** 2020-02-26

**Authors:** P. Brownell, F. Piccolo, F. Brims, R. Norman, D. Manners

**Affiliations:** 1 Department of Respiratory Medicine, St John of God Healthcare Midland Campus, Midland, Western Australia; 2 Department of Respiratory Medicine, Sir Charles Gairdner Hospital, Nedlands, Western Australia; 3 Curtin University Medical School, Bentley, Western Australia; 4 Curtin University School of Public Health, Bentley, Western Australia

**Correction to: BMC Pulmonary Med**

**https://doi.org/10.1186/s12890-020-1053-x**

Following publication of the original article [1], the authors flagged that the article had gone to publishing with errors in Tables 1, 2 and 3.

**Table 1:** Vignette variables and response options

Variable	Response options
Age (years)	50, 60, 70, 80
Gender	Male, female
Smoking status	Current lifelong smoker
	Quit smoking 5 years ago
	Smoked for about 10 years in their youth
	Never smoked
Symptoms	Cough and shortness of breath
	Haemoptysis
	Unintentional weight loss
	No respiratory symptoms – incidental finding on CT coronary angiogram
Lung nodule size (mm)	4, 5, 7, 9, 12, 19, 25, 30
Lung nodule location	Upper lobe, not upper lobe
Lung nodule spiculation	Yes, no
Recommendation from reporting radiologist	No recommendation
	Specialist respiratory review
	Urgent specialist respiratory review
	Repeat CT chest as per existing guidelines, probably in 3–6 months

**Table 2:** Case vignettes

Lung nodule case vignette	
Your patient is a 50 year old man. He is a current, lifelong smoker.	
He has a cough and worsening breathlessness.	
A CT of his chest shows a 4 mm left upper lobe nodule with spiculation.	
There is no recommendation provided by the reporting radiologist.	
*Does he need to be seen by a respiratory physician urgently (< 2 weeks) for suspected lung cancer?*	
Haemoptysis case vignette	
Your patient is a 60 year old man. He has never smoked.	
He has a small amount of haemoptysis.	
A CT of his chest is normal.	
There is no recommendation provided by the reporting radiologist.	
*Does he need to be seen by a respiratory physician urgently (< 2 weeks) for suspected lung cancer?*	
Lymphadenopathy case vignette	
Your patient is a 70 year old woman. She quit smoking 5 years ago.	
She has a cough and worsening breathlessness.	
A CT of her chest shows enlarged subcarinal and hilar lymph nodes without a lung lesion.	
There is no recommendation provided by the reporting radiologist.	
*Does she need to be seen by a respiratory physician urgently (< 2 weeks) for suspected lung cancer?*

**Table 3:** Participant demographic information, *n* = 152

Gender, n(%)	
Male	60 (39)
Female	92 (61)
Age, n(%)	
< 35 years	20 (13)
35–44 years	29 (19)
45–54 years	42 (28)
55–64 years	31 (20)
65–74 years	26 (17)
> 75 years	4 (3)
GP role, n(%)	
Vocationally registered	130 (86)
Non-vocationally registered	11 (7)
Registrar	9 (6)
Other	2 (1)
Years worked in general practice, n(%)	
< 5	24 (16)
5–9	23 (15)
10–19	30 (20)
20–29	29 (19)
30–39	28 (18)
> 40	18 (12)
Average number of hours worked per week, n(%)	
< 20	28 (19)
21–30	32 (21)
31–40	58 (38)
> 40	34 (22)
Location of primary practice, n(%)	
Capital city	70 (46)
Other metropolitan area*	28 (19)
Rural area^#^	40 (26)
Remote area^	14 (9)

*Population > 100,000 ^*#*^*Population 10,000–100,000* ^*^*^ *Population < 10,000*

The content of Table 2 had erroneously been replaced by a duplication of the content of Table 3, while the content of Table 1 had been erroneously replaced by the (correct) content of Table 2.

Furthermore, in the (non-PDF) version of Table 3 the top two rows were erroneously formatted in bold.

These errors have now been corrected in the original article.

Please also find the corrected tables below for reference.

The publisher apologizes for this technical error.
