# Supplementary material for: Does this lung nodule need urgent review? A discrete choice experiment of Australian general practitioners
Source: BMC Pulm Med. 2020 Jan 30;20:24. doi: 10.1186/s12890-020-1053-x (PMC6993503; doi:10.1186/s12890-020-1053-x)
Supplement: Supplementary file 1 — Additional file 1. Lung nodule case vignettes. [file 12890_2020_1053_MOESM1_ESM.docx]

**Supplement 1: Lung nodule case vignettes**

| Case number | Vignette |
| --- | --- |
| 1 | Your patient is a 50 year old man. He is a current, lifelong smoker.  He has a cough and worsening breathlessness.  A CT of his chest shows a 4mm left upper lobe nodule with spiculation.  There is no recommendation provided by the reporting radiologist.  Does he need to be seen by a respiratory physician urgently (<2 weeks) for suspected lung cancer? |
| 2 | Your patient is a 50 year old man. He smoked regularly until 5 years ago.  He has a small amount of haemoptysis.  A CT of his chest shows a 5mm right lower lobe nodule without spiculation.  The reporting radiologist recommends urgent respiratory review.  Does he need to be seen by a respiratory physician urgently (<2 weeks) for suspected lung cancer? |
| 3 | Your patient is a 50 year old man. He smoked for about 10 years in his youth.  He has no respiratory symptoms. The nodule was incidentally found on a CT coronary angiogram.  A CT of his chest shows a 7mm right upper lobe nodule with spiculation.  The reporting radiologist suggests repeating the CT chest as per existing guidelines, probably in 3 to 6 months.  Does he need to be seen by a respiratory physician urgently (<2 weeks) for suspected lung cancer? |
| 4 | Your patient is a 50 year old man. He has never smoked.  He has unintentionally lost 5kg of weight.  A CT of his chest shows a 9mm left lower lobe nodule without spiculation.  The reporting radiologist suggests respiratory specialist review.  Does he need to be seen by a respiratory physician urgently (<2 weeks) for suspected lung cancer? |
| 5 | Your patient is a 50 year old woman. She is a current lifelong smoker.  She has unintentionally lost 5kg of weight.  A CT of her chest shows a 12mm right upper lobe nodule without spiculation.  The reporting radiologist suggests repeating the CT chest as per existing guidelines, probably in 3 to 6 months.  Does she need to be seen by a respiratory physician urgently (<2 weeks) for suspected lung cancer? |
| 6 | Your patient is a 50 year old woman. She smoked regularly until 5 years ago.  She has no respiratory symptoms. The nodule was incidentally found on a CT coronary angiogram.  A CT of her chest shows a 19mm left lower lobe nodule with spiculation.  The reporting radiologist suggests respiratory specialist review.  Does she need to be seen by a respiratory physician urgently (<2 weeks) for suspected lung cancer? |
| 7 | Your patient is a 50 year old woman. She smoked for about 10 years in her youth.  She has a small amount of haemoptysis.  A CT of her chest shows a 25mm right upper lobe nodule without spiculation.  There is no recommendation provided by the reporting radiologist.  Does she need to be seen by a respiratory physician urgently (<2 weeks) for suspected lung cancer? |
| 8 | Your patient is a 50 year old woman. She has never smoked.  She has cough and worsening breathlessness.  A CT of her chest shows a 30mm right lower lobe nodule with spiculation.  The reporting radiologist suggests urgent respiratory specialist review.  Does she need to be seen by a respiratory physician urgently (<2 weeks) for suspected lung cancer? |
| 9 | Your patient is 60 year old man. He smoked for about 10 years in his youth.  He has no respiratory symptoms. The nodule was incidentally found on a CT coronary angiogram.  A CT of his chest shows a 9mm left lower lobe nodule without spiculation.  There is no recommendation provided by the reporting radiologist.  Does he need to be seen by a respiratory physician urgently (<2 weeks) for suspected lung cancer? |
| 10 | Your patient is a 60 year old man. He has never smoked.  He has unintentionally lost 5kg of weight.  A CT of his chest shows a 7mm right upper lobe nodule with spiculation.  The reporting radiologist recommends urgent respiratory specialist review.  Does he need to be seen by a respiratory physician urgently (<2 weeks) for suspected lung cancer? |
| 11 | Your patient is a 60 year old man. He is a current lifelong smoker.  He has cough and worsening breathlessness.  A CT of his chest shows a 5mm left lower lobe nodule without spiculation.  The reporting radiologist suggests repeating the CT chest as per existing guidelines, probably in 3 to 6 months.  Does he need to be seen by a respiratory physician urgently (<2 weeks) for suspected lung cancer? |
| 12 | Your patient is a 60 year old man. He smoked regularly until 5 years ago.  He has a small amount of haemoptysis.  A CT of his chest shows a 4mm right upper lobe nodule with spiculation.  The reporting radiologist suggests respiratory specialist review.  Does he need to be seen by a respiratory physician urgently (<2 weeks) for suspected lung cancer? |
| 13 | Your patient is a 60 year old woman. She smoked for about 10 years in her youth.  She has a small amount of haemoptysis.  A CT of her chest shows a 30mm right lower lobe nodule with spiculation.  The reporting radiologist suggests repeating the CT chest as per existing guidelines, probably in 3 to 6 months.  Does she need to be seen by a respiratory physician urgently (<2 weeks) for suspected lung cancer? |
| 14 | Your patient is a 60 year old woman. She has never smoked.  She has cough and worsening breathlessness.  A CT of her chest shows a 25mm left upper lobe nodule without spiculation.  The reporting radiologist suggests respiratory specialist review.  Does she need to be seen by a respiratory physician urgently (<2 weeks) for suspected lung cancer? |
| 15 | Your patient is a 60 year old woman. She is a current lifelong smoker.  She has unintentionally lost 5kg of weight.  A CT of her chest shows a 19mm left lower lobe nodule with spiculation.  There is no recommendation provided by the reporting radiologist.  Does she need to be seen by a respiratory physician urgently (<2 weeks) for suspected lung cancer? |
| 16 | Your patient is a 60 year old woman. She smoked regularly until 5 years ago.  She has no respiratory symptoms. The nodule was incidentally found on a CT coronary angiogram.  A CT of her chest shows a 12mm right upper lobe nodule without spiculation.  The reporting radiologist recommends urgent respiratory specialist review.  Does she need to be seen by a respiratory physician urgently (<2 weeks) for suspected lung cancer? |
| 17 | Your patient is a 70 year old man. He smoked for about 10 years in his youth.  He has cough and worsening breathlessness.  A CT of his chest shows a 12mm left lower lobe nodule with spiculation.  The reporting radiologist suggests respiratory specialist review.  Does he need to be seen by a respiratory physician urgently (<2 weeks) for suspected lung cancer? |
| 18 | Your patient is a 70 year old man. He has never smoked.  He has a small amount of haemoptysis.  A CT of his chest shows a 19mm right upper lobe nodule without spiculation.  The reporting radiologist suggests repeating the CT chest as per existing guidelines, probably in 3 to 6 months.  Does he need to be seen by a respiratory physician urgently (<2 weeks) for suspected lung cancer? |
| 19 | Your patient is a 70 year old man. He is a current lifelong smoker.  He has no respiratory symptoms. The nodule was incidentally found on a CT coronary angiogram.  A CT of his chest shows a 25mm left lower lobe nodule with spiculation.  The reporting radiologist suggests urgent respiratory specialist review.  Does he need to be seen by a respiratory physician urgently (<2 weeks) for suspected lung cancer? |
| 20 | Your patient is a 70 year old man. He smoked regularly until 5 years ago.  He has unintentionally lost 5kg of weight.  A CT of his chest shows a 30mm right upper lobe nodule without spiculation.  There is no recommendation provided by the reporting radiologist.  Does he need to be seen by a respiratory physician urgently (<2 weeks) for suspected lung cancer? |
| 21 | Your patient is a 70 year old woman. She smoked for about 10 years in her youth.  She has unintentionally lost 5kg of weight.  A CT of her chest shows a 4mm right lower lobe nodule without spiculation.  The reporting radiologist suggests urgent respiratory specialist review.  Does she need to be seen by a respiratory physician urgently (<2 weeks) for suspected lung cancer? |
| 22 | Your patient is a 70 year old woman. She has never smoked.  She has no respiratory symptoms. The nodule was incidentally found on a CT coronary angiogram.  A CT of her chest shows a 5mm left upper lobe nodule with spiculation.  There is no recommendation provided by the reporting radiologist.  Does she need to be seen by a respiratory physician urgently (<2 weeks) for suspected lung cancer? |
| 23 | Your patient is a 70 year old woman. She is a current lifelong smoker.  She has a small amount of haemoptysis.  A CT of her chest shows a 7mm left lower lobe nodule without spiculation.  The reporting radiologist suggests respiratory specialist review.  Does she need to be seen by a respiratory physician urgently (<2 weeks) for suspected lung cancer? |
| 24 | Your patient is a 70 year old woman. She smoked regularly until 5 years ago.  She has cough and worsening breathlessness.  A CT of her chest shows a 9mm right upper lobe nodule with spiculation.  The reporting radiologist suggests repeating the CT chest as per existing guidelines, probably in 3 to 6 months.  Does she need to be seen by a respiratory physician urgently (<2 weeks) for suspected lung cancer? |
| 25 | Your patient is an 80 year old man. He is a current lifelong smoker.  He has no respiratory symptoms. The nodule was incidentally found on a CT coronary angiogram.  A CT of his chest shows a 30mm left upper lobe nodule without spiculation.  The reporting radiologist suggests respiratory specialist review.  Does he need to be seen by a respiratory physician urgently (<2 weeks) for suspected lung cancer? |
| 26 | Your patient is an 80 year old man. He smoked regularly until 5 years ago.  He has unintentionally lost 5kg of weight.  A CT of his chest shows a 25mm left lower lobe nodule with spiculation.  The reporting radiologist suggests repeating the CT chest as per existing guidelines, probably in 3 to 6 months.  Does he need to be seen by a respiratory physician urgently (<2 weeks) for suspected lung cancer? |
| 27 | Your patient is an 80 year old man. He smoked for about 10 years in his youth.  He has cough and worsening breathlessness.  A CT of his chest shows a 19mm left upper lobe nodule without spiculation.  The reporting radiologist suggests urgent respiratory specialist review.  Does he need to be seen by a respiratory physician urgently (<2 weeks) for suspected lung cancer? |
| 28 | Your patient is an 80 year old man. He has never smoked.  He has a small amount of haemoptysis.  A CT of his chest shows a 12mm left lower lobe nodule with spiculation.  There is no recommendation provided by the reporting radiologist.  Does he need to be seen by a respiratory physician urgently (<2 weeks) for suspected lung cancer? |
| 29 | Your patient is an 80 year old woman. She is a current lifelong smoker.  She has a small amount of haemoptysis.  A CT of her chest shows a 9mm right upper lobe nodule with spiculation.  The reporting radiologist suggests urgent respiratory specialist review.  Does she need to be seen by a respiratory physician urgently (<2 weeks) for suspected lung cancer? |
| 30 | Your patient is an 80 year old woman. She smoked regularly until 5 years ago.  She has cough and worsening breathlessness.  A CT of her chest shows a 7mm left lower lobe nodule without spiculation.  There is no recommendation provided by the reporting radiologist.  Does she need to be seen by a respiratory physician urgently (<2 weeks) for suspected lung cancer? |
| 31 | Your patient is an 80 year old woman. She smoked for about 10 years in her youth.  She has unintentionally lost 5kg of weight.  A CT of her chest shows a 5mm right upper lobe nodule with spiculation.  The reporting radiologist suggests respiratory specialist review.  Does she need to be seen by a respiratory physician urgently (<2 weeks) for suspected lung cancer? |
| 32 | Your patient is an 80 year old woman. She has never smoked.  She has no respiratory symptoms. The nodule was incidentally found on a CT coronary angiogram.  A CT of her chest shows a 4mm left lower lobe nodule without spiculation.  The reporting radiologist suggests repeating the CT chest as per existing guidelines, probably in 3 to 6 months.  Does she need to be seen by a respiratory physician urgently (<2 weeks) for suspected lung cancer? |
